# Transcranial Doppler for internal carotid artery stenting: rationale and design of a randomized controlled trial

**DOI:** 10.3389/fneur.2026.1843954

**Published:** 2026-08-18

**Authors:** Yan Wang, Ying-Jia Wang, Lu Wang, Ting-Ting Wang, Hui-Sheng Chen

**Affiliations:** Department of Neurology, General Hospital of Northern Theater Command, Shenyang, China

**Keywords:** carotid artery stenting, severe internal carotid artery stenosis, sonothrombolysis, transcranial Doppler, trial protocol

## Abstract

**Background:**

Carotid artery stenting (CAS) is an effective revascularization procedure for internal carotid artery stenosis, but it is associated with a high incidence of perioperative cerebral embolism, with asymptomatic and symptomatic embolic events occurring in up to 40% of patients. Transcranial Doppler (TCD) has been shown to enhance fibrinolysis through sonothrombolysis and may reduce embolic burden during carotid interventions.

**Aims:**

To evaluate whether intraoperative and post-procedural TCD monitoring can reduce the number of new diffusion-weighted imaging (DWI) lesions after CAS in patients with severe internal carotid artery stenosis.

**Sample size estimates:**

A total of 232 patients are required to detect a 50% reduction in the median number of new DWI lesions (from 2.0 to 1.0) with 80% power and a two-sided *α* of 0.05, accounting for a 5% dropout rate.

**Design:**

TCD-ICAS is a prospective, randomized, single-center, blinded endpoint trial. Patients scheduled for CAS are randomized 1:1 to receive either TCD treatment (1.6 MHz, 0.274 MPa) from femoral artery puncture until 30–60 min after procedure completion plus standard care, or standard care alone.

**Outcomes:**

The primary efficacy endpoint is the number of new DWI lesions at 24 h post-randomization. The primary safety endpoint is intracranial hemorrhage within 24 h.

**Conclusion:**

The TCD-ICAS trial will assess the efficacy and safety of TCD as an adjuvant therapy to reduce cerebral embolic events in patients undergoing CAS.

**Clinical trial registration:**

Identifier (NCT07462546).

## Introduction

Carotid artery stenting (CAS) is a widely performed procedure for stroke prevention in patients with severe internal carotid artery stenosis (1–3). However, perioperative cerebral embolism remains a significant concern, with studies reporting new ischemic lesions on DWI in a substantial proportion of patients post-CAS (4).

Transcranial Doppler ultrasound is a non-invasive, real-time monitoring tool capable of detecting microembolic signals during carotid interventions (5). Beyond its diagnostic utility, emerging evidence suggests that TCD may exert therapeutic effects through ultrasound-enhanced thrombolysis (sonothrombolysis) (6–11). The SONOBUSTER trial demonstrated that TCD monitoring during carotid endarterectomy and stenting significantly reduced the incidence of new brain infarctions (12). Similarly, the SONOBIRDIE trial showed a reduction in cerebral embolism and stroke recurrence with TCD use during carotid endarterectomy (13). The proposed mechanism involves acoustic cavitation, which mechanically disrupts thrombus structure and enhances fibrinolytic drug penetration (14).

Building on these findings, we hypothesize that TCD application during and after CAS may reduce the number and volume of perioperative cerebral emboli, thereby decreasing the incidence of new ischemic lesions and improving clinical outcomes. The TCD-ICAS trial is designed to test this hypothesis in a randomized, controlled setting.

## Methods

### Study design

**Figure 1:**
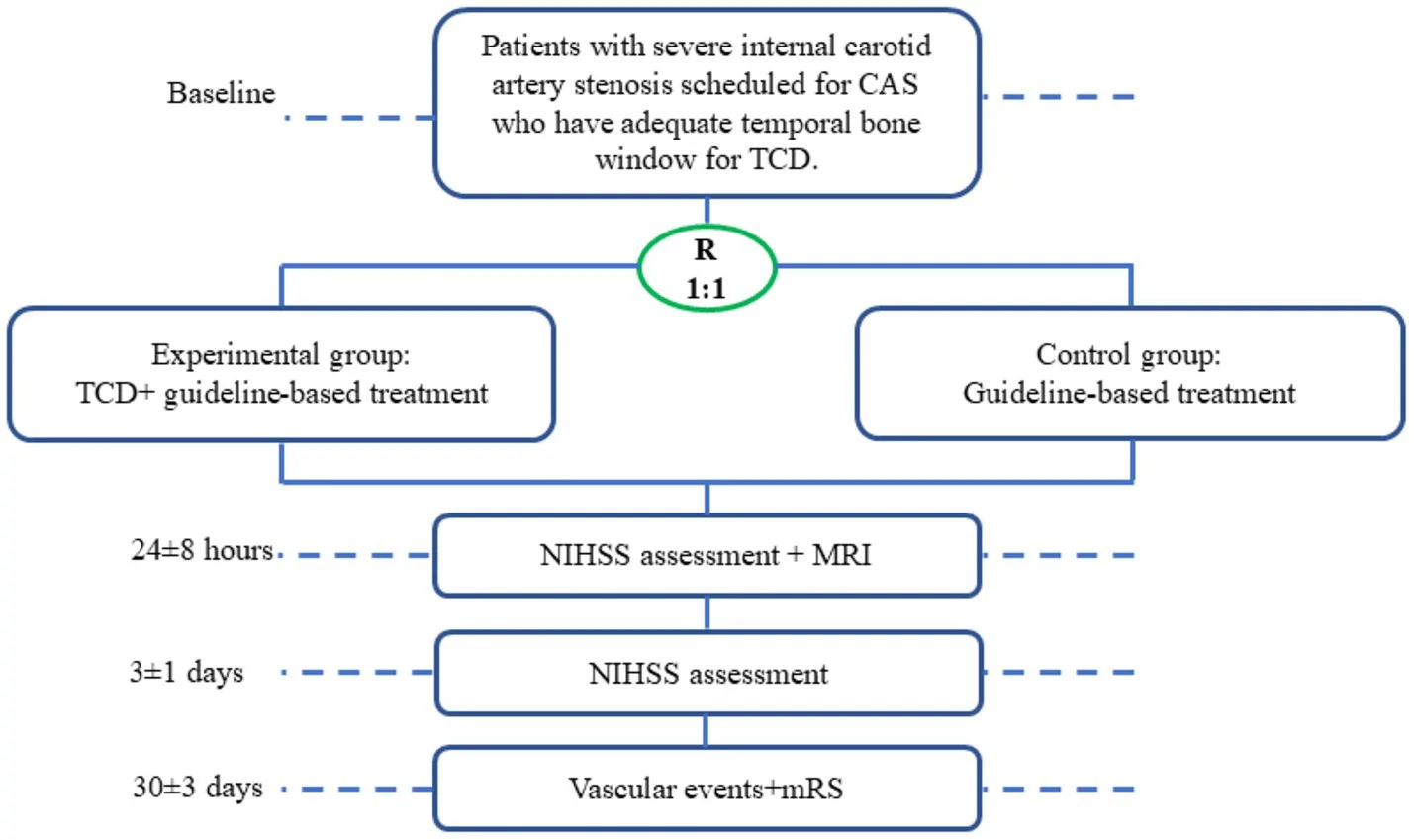
Study flowchart. CAS, carotid artery stenosis; TCD, Transcranial Doppler; NIHSS, National Institutes of Health Stroke Scale; MRI, magnetic resonance imaging; mRS, Modified Rankin Scale.

The TCD-ICAS trial is a prospective, randomized, single-center, blinded endpoint trial conducted at the General Hospital of Northern Theater Command, Shenyang, China. This study followed the Consolidated Standards of Reporting Trials (CONSORT) reporting guidelines. The trial flowchart is shown in Figure 1.

### Study population

In TCD-ICAS trial, eligible patients are adults with severe internal carotid artery stenosis scheduled for CAS who have adequate temporal bone window for TCD insonation. Key exclusion criteria include severe systemic infection or major organ dysfunction; intracranial hemorrhage within 90 days; severe coagulation disorders. Patients will be enrolled at General Hospital of Northern Theater Command between March 2026 to September 2027. The detailed inclusion and exclusion criteria are shown in Table 1.

**Table 1:** The inclusion/exclusion criteria.

The inclusion/exclusion criteria
Inclusion criteria
Age ≥ 40 years old;
Patients with severe stenosis of the internal carotid artery;
Planned for carotid artery stent;
Modified Rankin Scale score ≤ 2;
The subject has adequate temporal bone windows to undergo TCD, and the blood flow signal of the middle cerebral artery is detectable.;
Signed informed consent by patient or their legally authorized representative.
Exclusion criteria
Comorbid conditions with severe infections or severe diseases of the liver, kidney, hematopoietic system, endocrine system, etc.;
Occurrence of intracranial hemorrhage (cerebral parenchymal hemorrhage, subarachnoid hemorrhage, subdural/epidural hemorrhage) within 90 days prior to enrollment;
Severe hematological disorders or significant coagulation abnormalities;
Pregnant or breastfeeding women;
Participation in another clinical trial within the past 3 months or ongoing participation;
Any other circumstances deemed by the investigator as unsuitable for participation in this clinical study.*

* mainly include (1) contraindications to MRI; (2) life expectancy < 1 year due to advanced malignancy or end-stage organ failure; (3) severe psychiatric or cognitive disorders precluding informed consent or follow-up compliance; (4) anticipated poor adherence to scheduled follow-up assessments. Any other rare exclusion will be recorded with detailed justification in the case report form.

### Standard protocol approvals, registrations, and patient consents

The TCD-ICAS trial was prospectively registered on ClinicalTrials.gov (NCT07462546). Ethical approval for the study protocol and procedures was granted by the Ethics Committee of the General Hospital of Northern Theater Command (IRB: Y (2026)13). Written informed consent will be secured from every participant or their legally authorized representatives before inclusion into the trial.

### Randomization and intervention

Eligible patients are randomized 1:1 into the TCD group or control group via a computer-generated sequence, stratified by collateral status (poor vs. good) and age (≤65 vs. >65 years). In TCD group, patients will receive continuous TCD monitoring using a 1.6 MHz transducer insonating the ipsilateral middle cerebral artery (M1 segment, 55 mm depth) at 100% power output (0.274 MPa). The monitoring begins at femoral artery puncture and continues until 30–60 min after CAS completion. In the Control group, patients will only receive standard perioperative care without TCD monitoring. Both groups will receive guideline-based medical management. To ensure patient safety during prolonged continuous TCD insonation, we will continuously monitor the real-time Thermal Index (TI) and Mechanical Index (MI) displayed on the ultrasound system. For the 1.6 MHz transducer used in this protocol, TI is consistently < 1.0 and MI < 1.9, both well within diagnostic safety limits. The operator will also regularly ask the patient about any local discomfort or warmth during the procedure. Any reported symptoms will be documented as adverse events and reviewed by the Data and Safety Monitoring Committee.

### Outcome measures

Primary efficacy endpoint is number of new DWI lesions at 24 (±8) hours post-randomization. Secondary efficacy endpoints include total volume of new DWI lesions; number of lesions >0.5 mL; composite of ischemic stroke, transient ischemic attack (TIA), or death within 30 (±3) days.

Safety endpoints include intracranial hemorrhage within 24 (±8) hours; serious adverse events within 24 h.

### Follow-up procedure

All patients will undergo follow-up assessments at the following time points: before the CAS procedure, and at 24 ± 8 h, 3 ± 1 day, and 30 ± 3 days after randomization (see Table 2). Baseline evaluations will include collection of demographic information, laboratory tests, neuroimaging findings, and neurologic scores (NIHSS and mRS). Post-CAS assessments will consist of MRI (including DWI) and NIHSS at 24 ± 8 h, NIHSS at 3 ± 1 day, and mRS at 30 ± 3 days.

**Table 2:** Timing of all procedures.

Timepoint	Screening	0	24 ± 8 h	3 ± 1 days	30 ± 3 days
Eligibility screen	x				
Informed consent	x				
Randomization	x				
Head CT/MRI	x		x*		
NIHSS assessment	x	x	x	x	
mRS assessment	x				x
Medical history	x				
Population characteristics	x				
Vital signs	x	x	x	x	x
Blood routine	x				
Urinalysis	x				
Coagulation test	x				
Serum biochemistry	x				
ECG	x			x	
Concomitant medication		x	x	x	x
Adverse events		x	x	x	x
Recurrent stroke and other vascular events		x	x	x	x

CT indicates computed tomography; MRI, magnetic resonance imaging; NIHSS, National Institutes of Health Stroke Scale; mRS, modified Rankin Scale; ECG, electrocardiogram. * Diffusion-weighted imaging (DWI) is mandatory.

All clinical evaluations, including NIHSS and mRS, will be performed by qualified investigators who are blinded to TCD therapy following standardized protocol procedures. The primary efficacy outcome will also be assessed by independent neuroimaging reviewers who are blinded to treatment assignments.

Concomitant medications and adverse events occurring within 30 days after randomization will be recorded in detail by investigators and subsequently reviewed by certified adjudicators. The total duration of the study is estimated to be approximately 9 months.

### Quality control and data management

In this clinical trial, an independent Data and Safety Monitoring Committee (DSMC) has been established to ensure continuous monitoring of data safety, e.g., with a specific focus on endpoints such as hemorrhagic events and other severe adverse events (SAEs). Rules governing study termination or sample size modification will be pre-specified by the DSMC. Once follow-up has been completed for 50% of participants, an interim analysis will be carried out by an independent statistician affiliated with the DSMC, who will not be involved in the trial’s management. Based on the outcomes of the analysis, the DSMC will be authorized to make recommendations to the Steering Committee regarding adjustments to the sample size or early termination of the study. Additionally, neuroimaging data will be collected centrally and independently assessed by two neuroradiologists.

### Adverse event monitoring

An adverse event (AE) is defined as any adverse medical occurrence that takes place during the trial period. All information pertaining to AEs will be documented on the adverse event page(s) of the case report form (CRF). Additionally, the principal investigator (PI) will make a further assessment to determine whether an unexpected AE is associated with TCD.

### Sample size calculation

Based on published data (3), the median number of new DWI lesions in the control group was 2.0 (range 1.0–5.5). Fitting a negative binomial distribution to these data yielded an estimated mean of 2.3 and dispersion parameter k = 2.78. Assuming a 50% reduction (mean = 1.15) in the TCD group, with 80% power and a two-sided significance level of 0.05, a sample size of 110 patients per group is required. Inflating for 5% dropout gives 116 per group (total *N* = 232).

### Statistical analysis

Intention-to-treat analysis will be used. The primary endpoint will be analyzed using a negative-binomial generalized linear model with a log-link function. For volume of new cerebral infarcts, analysis of covariance (ANCOVA) will be used if normally distributed; log-transformation followed by ANCOVA or a non-parametric test (e.g., Mann–Whitney U test) will be applied for between-group comparison if the distribution is skewed. Number of new DWI infarcts >0.5 mL will be compared between groups using negative-binomial generalized linear model with a log-link function. For composite incidence of ischemic stroke, TIA, and death within 30 days, Kaplan–Meier curves will be plotted, and between-group event rates will be compared with the log-rank test. A Cox proportional-hazards regression model will be fitted to calculate the hazard ratio (HR) with 95% confidence interval (CI) for the TCD group versus the Control group.

All safety endpoints will be analyzed in the safety analysis set. Categorical variables (e.g., incidence of intracranial hemorrhage, serious adverse events) will be summarized as frequencies and percentages, with between-group comparisons performed using Fisher’s exact test. Continuous variables will be described as mean ± standard deviation or median (interquartile range) according to their distribution, and compared using t-tests or non-parametric tests as appropriate.

Exploratory analyses of multiple secondary endpoints will be conducted without adjustment for multiple comparisons; results are considered hypothesis-generating and should be interpreted with caution.

All statistical tests will be two-sided, with a significance level of *α* = 0.05; a *p*-value <0.05 will be considered statistically significant.

The primary outcome will further be analyzed according to subgroups: age (<60 years vs. ≥ 60 years), sex (female vs. male), history of diabetes (yes vs. no), history of hypertension (yes vs. no), degree of index vessel stenosis (70–84% vs. 85–99%), collateral status (poor vs. good). Differences in the primary outcome across the aforementioned subgroups will be evaluated by assessing the interaction effect between the pre-specified baseline variables and the primary outcome.

### Study organization and funding

The Trial Steering Committee (TSC), comprising external scientific advisors, will conduct regular oversight of the study conduct and data management. The TSC will hold meetings every six months, either via teleconference or in person, to provide recommendations pertaining to patient enrollment and trial implementation. This trial is sponsored and supported by the Cerebrovascular Disease Collaboration & Innovation Alliance (CDCIA) of Liaoning Province.

### Current status

At the time of this manuscript’s initial submission, the patient’s recruitment has commenced, and 12 patients are enrolled. Recruitment will remain open until the target sample size is achieved, with completion anticipated no later than December 2026.

## Discussion

The TCD-ICAS trial addresses a critical and persistent clinical challenge in neurointervention: reducing cerebral embolic burden during CAS. Despite advances in embolic protection devices, peri-procedural microembolization remains a key contributor to postoperative ischemic lesions and associated cognitive decline. This trial proposes a novel, non-pharmacological strategy by employing TCD monitoring not only as a diagnostic tool but as an active therapeutic modality throughout the procedure and into the immediate postoperative window. The underlying hypothesis leverages the sonothrombolytic effect of TCD, whereby targeted ultrasound energy may promote the disaggregation and clearance of microemboli, thereby potentially mitigating silent cerebral infarction and improving neurological outcomes.

This investigation is grounded in and extends prior mechanistic and clinical research. It directly builds upon the promising results of trials such as SONOBUSTER and SONOBIRDIE, which provided preliminary evidence for the feasibility and efficacy of TCD in enhancing clot lysis and reducing embolic signals during carotid interventions (12, 13). However, TCD-ICAS distinguishes itself by focusing exclusively on the CAS population—a group at high risk for thromboembolic complications—and by implementing a standardized, prolonged sonication protocol. This extended therapeutic window, encompassing both the intraoperative and early recovery phases, is designed to address the continued embolic risk that persists after stent deployment, potentially offering a more comprehensive protective effect than shorter intraoperative applications alone.

Given the enough eligible patients in our center, the single-center design was used in this trial, which also ensures rigorous standardization and consistency in both TCD application parameters and endovascular technique. Nevertheless, we acknowledge that this design may limit the immediate generalizability of the findings to centers with different procedural protocols. A key methodological strength is the implementation of blinded, centralized adjudication for the primary imaging endpoint (new DWI lesions), which effectively minimizes assessment bias and strengthens the validity of the outcome evaluation.

The TCD-ICAS trial would position TCD not merely as a monitoring device but as a low-cost, non-invasive, and readily available therapeutic adjunct to CAS. Integrating TCD into standard CAS protocols could enhance the procedure’s safety profile, potentially reducing the incidence of peri-procedural stroke. Future research steps, contingent on positive outcomes, would include larger multicenter validation studies, investigations into optimal sonication parameters, and analyses of long-term cognitive outcomes and cost-effectiveness to firmly establish its role in routine cerebrovascular revascularization care.

In summary, the TCD-ICAS trial represents a targeted translational effort to address the issue of peri-procedural cerebral embolization during CAS. By repurposing a widely available monitoring tool into an active therapeutic strategy, this study investigates a practical and potentially cost-effective adjunct to current practice. This work could provide evidence for TCD as a valuable non-invasive intervention to enhance the safety of carotid stenting.
